# Supplementary material for: Clinical Recommendations to Manage Gastrointestinal Adverse Events in Patients Treated with Glp-1 Receptor Agonists: A Multidisciplinary Expert Consensus
Source: J Clin Med. 2022 Dec 24;12(1):145. doi: 10.3390/jcm12010145 (PMC9821052; doi:10.3390/jcm12010145)
Supplement: Supplementary file 1 [file jcm-12-00145-s001.zip › jcm-2088871-SI.pdf]

Supplementary Table S1. Characteristics of the currently commercialized GLP-1 RAs with indications for T2D or obesity

Active ingredient	Trade name	Indication	RD (mg)	Administration	Posology
Semaglutide[1]	Ozempic®	Adults with insufficiently controlled T2D as an adjunct to diet and exercise<br>● as monotherapy if metformin is inappropriate due to intolerance or contraindications<br>● in addition to other medicinal products for the treatment of diabetes	0.5, 1.0, 2.0	s.c.	Once weekly
Semaglutide[2]	Rybelsus®	Adults with insufficiently controlled T2D as an adjunct to diet and exercise<br>● as monotherapy if metformin is inappropriate due to intolerance or contraindications<br>● in addition to other medicinal products for the treatment of diabetes	7, 14	p.o.	QD
Semaglutide[3]	Wegovy®	Adults with BMI ≥30 kg/m² or ≥27 kg/m² to <30 kg/m² and ≥1 weight-related comorbidity* as an adjunct to diet and exercise	2.4	s.c.	Once weekly
Liraglutide[4]	Victoza®	Adults, adolescents and ≥ 10 y.o. children with insufficiently controlled T2D as an adjunct to diet and exercise<br>● as monotherapy if metformin is inappropriate due to intolerance or contraindications<br>● in addition to other medicinal products for the treatment of diabetes	1.2, 1.8	s.c.	QD
Liraglutide[5]	Saxenda®	Adults with BMI ≥30 kg/m² or ≥27 kg/m² to <30 kg/m² and ≥1 weight-related comorbidity† as an adjunct to diet and exercise<br>Adolescents (≥12 y.o.) with BMI corresponding to ≥30 kg/m² for adults by IOTF BMI cut-off points and body weight >60 kg	3	s.c.	QD
Dulaglutide[6]	Trulicity®	Adults with insufficiently controlled T2D as an adjunct to diet and exercise<br>● as monotherapy if metformin is inappropriate due to intolerance or contraindications<br>● in addition to other medicinal products for the treatment of diabetes	0.75‡<br>1.5§	s.c.	Once weekly

Exenatide[7]	Bydureon®	≥18 y.o. adults with T2D in combination with other glucose-lowering medicinal products including basal insulin, when the therapy in use, together with diet and exercise, does not provide adequate glycaemic control.	2	s.c.	Once weekly
Exenatide[8]	Byetta®	● Adults with T2D in combination with: metformin; sulphonylureas; thiazolidinediones; metformin and a sulphonylurea; metformin and a thiazolidinedione, when adequate glycaemic control has not been achieved on maximally tolerated doses of these therapies<br>● Adults with T2D, as adjunctive therapy to basal insulin with or without metformin and/or pioglitazone when adequate glycaemic control has not been achieved with such treatments	0.010	s.c.	BID
Lixisenatide[9]	Lyxumia®	Adults with T2D in combination with oral glucose-lowering medicinal products and/or basal insulin when these, together, with diet and exercise, do not provide adequate glycaemic control	0.020	s.c.	QD

1. https://www.ema.europa.eu/en/documents/product-information/ozempic-epar-product-information_en.pdf

2. https://www.ema.europa.eu/en/documents/product-information/rybelsus-epar-product-information_en.pdf

3. https://www.ema.europa.eu/en/documents/product-information/wegovy-epar-product-information_en.pdf

4. https://www.ema.europa.eu/en/documents/product-information/victoza-epar-product-information_en.pdf

5. https://www.ema.europa.eu/en/documents/product-information/saxenda-epar-product-information_en.pdf

6. https://www.ema.europa.eu/en/documents/product-information/trulicity-epar-product-information_en.pdf

7. https://www.ema.europa.eu/en/documents/product-information/bydureon-epar-product-information_en.pdf

8. https://www.ema.europa.eu/en/documents/product-information/byetta-epar-product-information_en.pdf

9. https://www.ema.europa.eu/en/documents/product-information/lyxumia-epar-product-information_en.pdf

*Prediabetes or T2D, hypertension, dyslipidaemia, obstructive sleep apnoea or cardiovascular disease. †Prediabetes or T2D, hypertension, dyslipidaemia, obstructive sleep apnoea. ‡Monotherapy. §Add-on therapy. BID, twice a day; BMI, body mass index; GLP-1 RA, GLP-1 receptor agonist; IOTF, International Obesity Task Force; p.o., oral; s.c., subcutaneous; QD, once a day; RD, recommended dose; T2D, type 2 diabetes mellitus; y.o., years old.

Supplementary Table S2. Combinations of keywords used to perform a literature search in the PubMed database regarding management of AEs associated with GLP-1 RA use

Keyword combination	Retrieved results (n)
Glucagon-Like Peptide-1 receptor agonist*[Title] and practic*[Title]	1
Glucagon-Like Peptide-1 receptor agonist*[Title] and manag*[Title]	0
Glucagon-Like Peptide-1 receptor agonist*[Title] and adverse event*[Title]	0
Glucagon-Like Peptide-1 receptor agonist*[Title] and safe*[Title]	28
Glucagon-Like Peptide-1 receptor agonist*[Title] and real-world[Title]	22
GLP-1 receptor agonist*[Title] and practic*[Title]	0
GLP-1 receptor agonist*[Title] and manag*[Title]	0
GLP-1 receptor agonist*[Title] and adverse event*[Title]	0
GLP-1 receptor agonist*[Title] and safe*[Title]	18
GLP-1 receptor agonist*[Title] and real-world[Title]	6
Semaglutide[Title] and practic*[Title]	0
Semaglutide[Title] and manag*[Title]	0
Semaglutide[Title] and adverse event*[Title]	0
Semaglutide[Title] and safe*[Title]	36
Semaglutide[Title] and real-world[Title]	17
Semaglutide[Title] and SUSTAIN[Title]	36
Semaglutide[Title] and STEP[Title]	9
Semaglutide[Title] and PIONEER[Title]	31
Liraglutide[Title] and practic*[Title]	0
Liraglutide[Title] and manag*[Title]	0
Liraglutide[Title] and adverse event*[Title]	1
Liraglutide[Title] and safe*[Title]	60
Liraglutide[Title] and real-world[Title]	52
Liraglutide[Title] and LEAD[Title]	16
Liraglutide[Title] and SCALE[Title]	13
Dulaglutide[Title] and practic*[Title]	0
Dulaglutide[Title] and manag*[Title]	0
Dulaglutide[Title] and adverse event*[Title]	0
Dulaglutide[Title] and safe*[Title]	41
Dulaglutide[Title] and real-world[Title]	21
Dulaglutide[Title] and AWARD[Title]	41
Exenatide[Title] and practic*[Title]	0
Exenatide[Title] and manag*[Title]	0
Exenatide[Title] and adverse event*[Title]	1
Exenatide[Title] and safe*[Title]	38
Exenatide[Title] and real-world[Title]	18

Exenatide[Title] and DURATION[Title]	29
Lixisenatide[Title] and practic*[Title]	0
Lixisenatide[Title] and manag*[Title]	0
Lixisenatide[Title] and adverse event*[Title]	0
Lixisenatide[Title] and safe*[Title]	33
Lixisenatide[Title] and real-world[Title]	5
Lixisenatide[Title] and GETGOAL[Title]	23

AEs, adverse events; GLP-1 RA, glucagon-like peptide-1 receptor agonist.
